# Heterogeneous Nuclear Ribonucleoprotein A3 Is the Liver Nuclear Protein Binding to Age Related Increase Element RNA of the Factor IX Gene

**DOI:** 10.1371/journal.pone.0012971

**Published:** 2010-09-24

**Authors:** Toshiyuki Hamada, Sumiko Kurachi, Kotoku Kurachi

**Affiliations:** Age Dimension Research Center, National Institute of Advanced Industrial Science and Technology, Tsukuba, Ibaraki, Japan; The Research Institute for Children, United States of America

## Abstract

**Background:**

In the ASE/AIE-mediated genetic mechanism for age-related gene regulation, a recently identified age-related homeostasis mechanism, two genetic elements, ASE (age-related stability element) and AIE (age-related increase element as a stem-loop forming RNA), play critical roles in producing specific age-related expression patterns of genes.

**Principal Finding:**

We successfully identified heterogeneous nuclear ribonucleoprotein A3 (hnRNP A3) as a major mouse liver nuclear protein binding to the AIE-derived RNAs of human factor IX (hFIX) as well as mouse factor IX (mFIX) genes. HnRNP A3 bound to the AIE RNA was not phosphorylated at its Ser^359^, while hnRNP A3 in the mouse liver nuclear extracts was a mixture of phosphorylated and unphosphorylated Ser^359^. HepG2 cells engineered to express recombinant hFIX transduced with adenoviral vectors harboring an effective siRNA against hnRNP A3 resulted in a substantial reduction in hFIX expression only in the cells carrying a hFIX expression vector with AIE, but not in the cells carrying a hFIX expression vector without AIE. The nuclear hnRNP A3 protein level in the mouse liver gradually increased with age, while its mRNA level stayed age-stable.

**Conclusions:**

We identified hnRNP A3 as a major liver nuclear protein binding to FIX-AIE RNA. This protein plays a critical role in age-related gene expression, likely through an as yet unidentified epigenetic mechanism. The present study assigned a novel functional role to hnRNP A3 in age-related regulation of gene expression, opening up a new avenue for studying age-related homeostasis and underlying molecular mechanisms.

## Introduction

We previously reported the first molecular mechanism of age-related homeostasis, the ASE/AIE-mediated genetic mechanism for age-related gene expression [1]–[3]. In this mechanism, two genetic elements, designated as ASE (age-related stability element) and AIE (age-related increase element), play essential roles for producing age-related stable and age-related increase profiles of a gene expression, respectively. ASE functions independently of AIE, while AIE requires ASE for generating a fully age-related increase pattern of gene expression. More recently, we further demonstrated the clinical relevance of this mechanism by proving its critical role in the pathological mechanism of hemophilia B Leyden, a subfamily of hemophilia B showing unique puberty-onset amelioration, and showed that this mechanism actually functions as a puberty-onset gene switch mechanism [4]. A specific liver protein binding to the functional ASE (only G/CAGGAAG out of all heptanucleotide combinations) was identified as Ets1 [4].

AIE originally identified in the hFIX gene has a 102 base pair (bp) stretch of dinucleotide repeats (rich in AT, GT and CA) in the middle of 3′-untranslated region (3′-UTR), which potentially forms and functions as a stem-loop RNA structure (hereafter referred as hFIX-AIE RNA) after the gene is transcribed [1], [2], [5]. The mFIX gene also shows an age-related increase expression pattern [1], and has a stretch of dinucleotide repeats (rich in AT) of about a 50 bp in the middle of 3′-UTR, potentially forming a stem-loop RNA structure (referred as mFIX-AIE RNA) after gene transcription [2]. Both hFIX-AIE RNA and mFIX-AIE RNA function equally well in a position-dependent and orientation-independent manner for producing an age-related increase pattern of gene expression as tested with the human protein C gene, lacking AIE [2].

In this report, we describe identification of hnRNP A3 as the mouse liver nuclear protein, which specifically bind to hFIX-AIE RNA as well as to mFIX-AIE RNA, its phosphorylation status in binding to AIE RNA, functional characterization analyzed by siRNA and age-related expression profiles of its gene and protein, thus assigning a novel functional role to this protein.

## Results

### Specific binding of liver nuclear protein(s) to hFIX-AIE RNA and mFIX-AIE RNA

**Figure 1:**
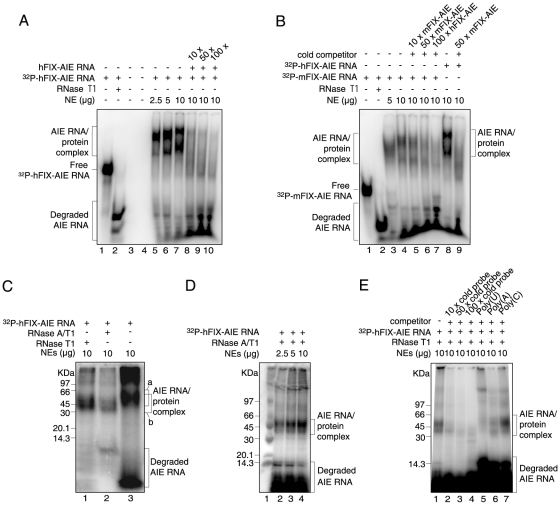
EMSAs and SDS-PAGE analyses of ^32^P-hFIX-AIE RNA/nuclear protein and ^32^P-mFIX-AIE RNA/nuclear protein complexes. **A.** EMSAs of ^32^P-hFIX-AIE RNA with liver NEs. Various conditions tested are shown at the top. Brackets on the left indicate hFIX-AIE RNA probe-protein complexes and degraded ^32^P-hFIX-AIE RNA as shown. The position of free ^32^P-hFIX-AIE RNA is shown with a short horizontal bar on the left. Lanes 1, 2 and 5–10 contain ^32^P-hFIX-AIE RNA. Lane 2, treated with RNase T1; lanes 3 and 4, null controls; lanes 5–7, with increasing amounts of liver NEs; lanes 8–10, with NEs (10 µg) and increasing amounts of cold hFIX-AIE RNA competitor. **B.** EMSAs of ^32^P-mFIX-AIE or ^32^P-hFIX-AIE RNA with liver NEs. Lanes 1–7 contains ^32^P-mFIX-AIE RNA, while lanes 8 and 9 contain ^32^P-hFIX-AIE RNA. Positions of the AIE RNA probe-protein complex, free AIE RNA probe and degraded probe are similarly shown as in A. Lane 1, without NEs; lane 2, treated with RNase T1; lanes 3 and 4, with increasing amounts of NEs; lanes 5 and 6, with NEs (10 µg) and increasing amounts of cold mFIX-AIE RNA competitor; lane 7, with NEs (10 µg) and cold hFIX-AIE RNA; lane 8, with NEs (10 µg); lane 9, with NEs (10 µg) and cold mFIX-AIE RNA. **C**. SDS-PAGE analysis of UV cross-linked ^32^P-hFIX-AIE RNA/nuclear protein complex treated with RNase T1 (lane 1), RNase A/T1 (lane 2) and no RNase treatment (lane 3). Brackets a and b represent the positions of AIE RNA-protein complex without and with RNase-treatment, respectively. Size marker positions are shown on the left. **D**. SDS-PAGE analysis of UV cross-linked and RNase T1-treated ^32^P-hFIX-AIE RNA incubated with increasing amounts of NEs. Positions for the AIE RNA/protein complex and degraded ^32^P-hFIX-AIE RNA are shown with brackets on the right. Lane 1, protein size marker with sizes shown on the left. **E.** SDS-PAGE analysis after competitive EMSAs of ^32^P-hFIX-AIE RNA with cold hFIX-AIE RNA (lanes 2–4), and with poly(U), poly(A) and poly(C) (lanes 5–6). Other conditions are similar to D.

As shown in Figure 1A, electrophoretic mobility shift assay (EMSA) of mouse liver nuclear extracts (NEs) with ^32^P-hFIX-AIE RNA resulted in two major mobility shifted bands with increasing amounts of NEs (lanes 5–7), suggesting that hFIX-AIE RNA specifically bind at least two nuclear proteins. Intensities of these bands drastically reduced with addition of excess amounts of non-radiolabeled hFIX-AIE RNA (Fig. 1A, lanes 8–10), confirming the specificity of protein binding to the hFIX-AIE RNA probe. As shown in Fig. 1B, ^32^P-mFIX-AIE RNA probe also produced similar two shifted bands with increasing amounts of NEs (lanes 3 and 4), and their intensities again efficiently reduced by addition of non-radiolabled mFIX-AIE RNA probe (lanes 5 and 6) as well as of non-radiolabled hFIX-AIE RNA (lane 7). The shifted bands generated with ^32^P-hFIX-AIE RNA (lane 8) were also efficiently reduced with addition of non-radiolabled mFIX-AIE RNA (lane 9), indicating that both hFIX-AIE RNA and mFIX-AIE RNA likely bind same nuclear proteins. Reflecting the smaller size of mFIX-AIE RNA (50 nucleoside residues) than that of hFIX-AIE RNA (149 nucleoside residues) used, the band shifts produced with ^32^P-mFIX-AIE RNA (Fig. 1B, lanes 3 and 4) were smaller than those observed with ^32^P-hFIX-AIE RNA (lane 8). When hFIX-AIE RNA probe/protein complexes were UV cross-linked, two bands of approximate 44 kDa (major) and 50 kDa (minor) shown with bracket a were observed in sodium dodecylsulfate polyacrylamide gel electrophoresis (SDS-PAGE), and with subsequent RNase treatments, their sizes lowered to about 40 and 45 kDa shown with bracket b, respectively, presumably due to a loss of the RNase-accessible portions of RNA fragment (Fig. 1C). Similar reductions in size were also seen with UV cross-linked and RNase-treated ^32^P-mFIX-AIE RNA/nuclear protein complex (data not shown). Two major mobility shifted bands observed for the UV cross-linked and RNase-treated hFIX-AIE RNA increased their band intensities with increasing amounts of NEs used (Fig. 1D, lanes 2–4), supporting the specific nuclear protein binding to ^32^P-hFIX-AIE RNA shown by band-shift assays (Fig. 1A, lanes 5–7). Competitive reductions in the intensity of ^32^P-hFIX-AIE RNA/nuclear protein complex bands observed with addition of the cold hFIX-AIE RNA probe (Fig. 1A, lanes 8–10) were maintained through UV cross-linking/RNase-treatment processes, indicating that the complex formation is specific and not a false event (Fig. 1C, D).

In a separate series of experiment with non-specific homo-poly nucleosides, homo-poly(C), showed no significant competition in RNA EMSA, while homo-poly(U) and homo-poly(A) showed significant competitions on formation of ^32^P-hFIX-AIE RNA/nuclear protein complex. The competition, however, was significantly weaker than that with cold hFIX-AIE RNA (Fig. 1E).

### Identification of hnRNP A3 as the liver nuclear protein specifically binding to FIX-AIE RNA

**Figure 2:**
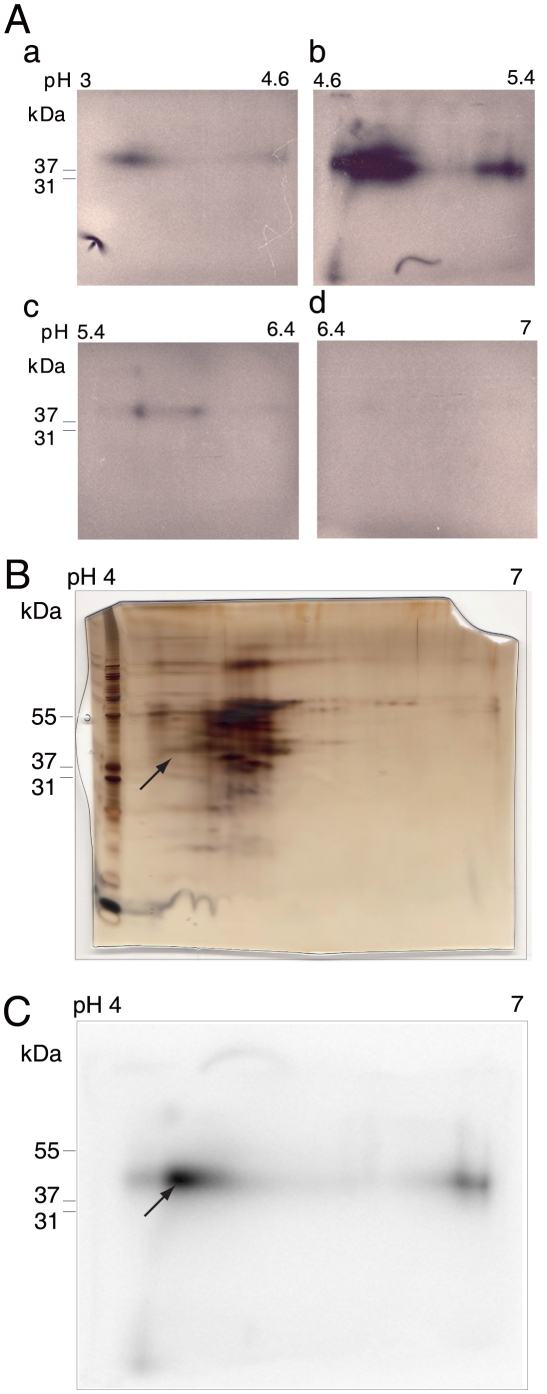
2DE analysis of UV cross-linked/RNase-treated ^32^P-hFIX-AIE RNA/liver nuclear protein complex and its autoradiography. **A**. Analytical scale 2DE analyses of the solution-phase IEF chamber solutions containing UV cross-linked/RNase-treated ^32^P-hFIX-AIE RNA/protein complexes. Chamber solutions of pH 3–4.6, pH 4.6–5.4, pH 5.4–6.4 and pH 6.4–7.0 zones were subjected to analytical 2DEs using immobilized pH 4–7 gradient gels for IEF and 4–12% gradient gel SDS-PAGE, and the results are shown in panels a, b, c and d, respectively. **B**. Silver-stained 2DE gel of UV cross-linked and RNase-treated ^32^P-hFIX-AIE RNA/nuclear protein complexes concentrated to pH 3–4.6 and pH 4.6–5.4 chambers in the solution phase IEF. Molecular size marker proteins are shown on the left edge of the gel with their sizes. Arrow indicates the protein spot position matching with the center of the major radioactive spot detected by autoradiography. **C**. Autoradiogram of the gel shown in B. Arrow indicates the center of the major radioactive spot, and the gel recovered from this center area was subjected to subsequent MALDI-TOF/MS analysis.

**Figure 3:**
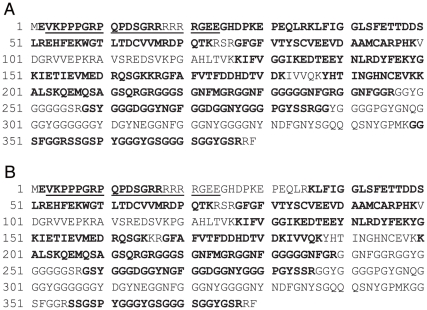
Amino acid sequences of mouse hnRNP A3 in complex with hFIX-AIE RNA and mFIX-AIE RNA. Amino acid sequences of mouse liver hnRNP A3 (isoform hnRNP A3a) bound with hFIX-AIE RNA (**A**) and mFIX-AIE RNA (**B**), identified by MALDI-TOF/MS and subsequent Mascot search analyses, are shown in bald letters of its known complete sequence [6]. Underlines indicate the sequence region absent in hnRNP A3b, a short isoform of hnRNP A3 generated by an alternative splicing [6]. In the present mass-spectrometric analyses, no discrimination between hnRNP A3a and hnRNP A3b was made. Domain structures in analogy to other known hnRNP family proteins include RRM 1 (RNA recognition motif: aa 35–118), RRM 2 (RNA recognition motif: aa 126–205) and RGG motif (aa 211–379) [6]–[8].

A sufficient amount of ^32^P-hFIX-AIE RNA/protein complex required for preparative solution-phase isoelectric focusing (IEF) was obtained by repeating the procedures of RNA EMSA with the ^32^P-hFIX-AIE RNA probe, UV-irradiation and RNase digestion of the ^32^P-hFIX-AIE RNA probe/protein complex extracted, SDS-PAGE separation, subsequent excision of the radio-active gel area corresponding to 30 kDa–60 kDa and extraction of the treated complex by electro-elution. Two dimensional electrophoresis (2DE) analyses of the complex samples showed that the solution-phase IEF efficiently concentrated the most ^32^P-hFIX-AIE RNA/protein complex to pH 4.6–5.4 zone (Fig. 2A, b), a much less, but significant amount to pH 3–4.6 zone (Fig. 2A, a), and only very low or negligible amounts to pH 5.4–6.4 and pH 6.4–7.0 zones (Fig. 2A, c and d, respectively**)**. Subsequent 2DE (Fig. 2B) and autoradiography (Fig. 2C) of the pooled solutions of pH 3–4.6 and pH 4.6–5.4 zones identified distinct multiple radioactive gel spots with the major one centered at pH 4.5 and 43 kDa in molecular size. Matrix-assisted laser desorption/ionization-time-of-flight mass-spectrometer (MALDI-TOF/MS) and Peptide mass fingerprint (PMF) analyses of the proteins extracted from the major radioactive gel spot reproducibly identified mouse hnRNP A3 (UniProtKB/Swiss-Prot accession number: Q8BG05) as the only protein component (p<0.05) with a high Mascot score of 152 (Fig. 3A). Similar analyses of ^32^P-mFIX-AIE RNA probe/nuclear protein complexes also identified hnRNP A3 as the only protein component with a high Mascot score of 117 (Fig. 3B). In these analyses, both two known hnRNP A3 isoforms, hnRNP A3a (full-length form) and hnRNP A3b (a short form due to an alternative splicing) [6]–[8] were detected together (here, their names were redefined to eliminate previously existing minor ambiguities). Four independent experiments, two with ^32^P-hFIX-AIE RNA and two with ^32^P-mFIX-AIE RNA, reproducibly identified hnRNP A3 as the nuclear protein binding to these AIE RNA probes. In one of the two experiments with hFIX-AIE RNA, however, hnRNP A2/B1 was also detected (UniProtKB accession number: O88569), although not at very significant level (Mascot score 53, p = 0.072) in addition to hnRNP A3. Since no hnRNP A2/B1 as well as other proteins were detected in all other three independent experiments with both hFIX-AIE RNA and mFIX-AIE RNA, hnRNP A3 was determined to be a major AIE RNA binding nuclear protein. In 2DE, the FIX-AIE RNA/hnRNP A3 complex migrated to pI3-5.4 shifting from the expected pI7-9 for the free form of hnRNP A3 [9]–[11], apparently due to the bound RNA probe.

**Figure 4:**
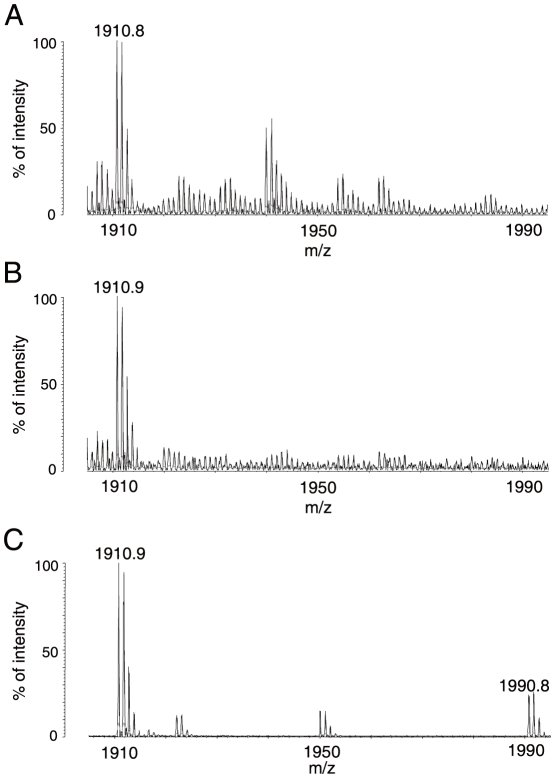
MALDI-TOF/MS spectra of peptides generated from mouse hnRNP A3 in complex with hnFIX-AIE RNA and mFIX-AIE RNA, and with no AIE RNA. **A.** Cropped mass spectra of the 2DE protein spot containing the UV-irradiated and RNase-treated ^32^P-hFIX-AIE RNA/hnRNP A3 complex. Mass-spectrum m/z 1910.8 corresponding to a tryptic peptide of hnRNP A3 (aa 356 through 377) with Ser^359^ unphosphorylated was reproducibly detected, while mass-spectrum m/z 1990.8 corresponding to the same peptide with phosphorylated Ser^359^ was not detected. **B.** Cropped mass spectra of the 2DE protein spot containing the UV-irradiated and RNase-treated ^32^P-mFIX-AIE RNA/hnRNP A3 complex. Experimental conditions used and observations made were similar to those described in A. **C.** Cropped mass spectra of one of protein spots separated in 2DE of liver NEs (free of hnRNP-AIE RNA). Both spectra m/z 1910.9 and m/z 1990.8 were reproducibly detected.

In MALDI-TOF/MS analyses carried out, a mass-spectrum m/z 1910.8 or 1910.9 corresponding to the peptide spanning amino acid (aa) 356–377 (SSGSPYGGGYGSGGGSGGYGSR; see Fig. 3) with its Ser^359^ (Fig. S1) unphosphorylated was reproducibly detected, while a mass-spectrum m/z 1990.8 corresponding to the peptide with the Ser^359^ residue phosphorylated (Fig. S1), was always absent (Fig. 4A and B). The aa numbering system used here included the first methionine residue as aa 1 so that the numbering is consistent with that customarily used for this protein [6].

### Phosphorylation status at Ser^359^ of hnRNP A3 protein

2DE and subsequent MALDI-TOF/TOF/MS (MALDI-TOF2) analyses of liver NEs obtained from mice at 3 months of age identified at least 19 protein spots to contain hnRNP A3 (data not shown). By definition of Mascot scores ≥53 being significant, 14 and 5 of these spots were found to contain only hnRNP A3 protein and mixtures of proteins including hnRNP A3, respectively. Four of the 14 hnRNP A3 single protein spots contained hnRNP A3 with its Ser^359^ phosphorylated (Fig. S1), while others contained hnRNP A3 with its Ser^359^ unphosphorylated. As shown in Fig. 4C, however, MALDI-TOF2 analyses of these four hnRNP A3 protein spots reproducibly detected both mass-spectra m/z 1910.9 and m/z 1990.8, indicating that Ser^359^ in the above mentioned peptide (aa 356–377) is in a mixed state of unphosphorylated and phosphorylated. Liver nuclear proteins prepared from 6 month-old mice had the same number of protein spots of hnRNP A3 with their Ser^359^ residues phosphorylated in a similar 2DE separation pattern found for 3 month-old animals, while liver nuclear proteins prepared from animals at 18 and 21 months of age showed two more hnRNP A3 protein spots with their Ser^359^ phoshorylated (data not shown). Both mass-spectra m/z 1910.9 and m/z 1990.8 were always observed with hnRNP A3 protein obtained from these spots. In the present study, Ser^359^ was most candidate phosphorylation site (Fig. S1) and no evidence for possible phosphorylation of aa residues other than Ser^359^. Furthermore, 2DE analyses of the cytosol proteins were unable to detect any hnRNP A3 containing protein spots, likely due to its very low concentration in the liver cell cytosol fraction.

### Effects of siRNA against hnRNP A3 on hFIX minigene expression in HepG2 cells

**Figure 5:**
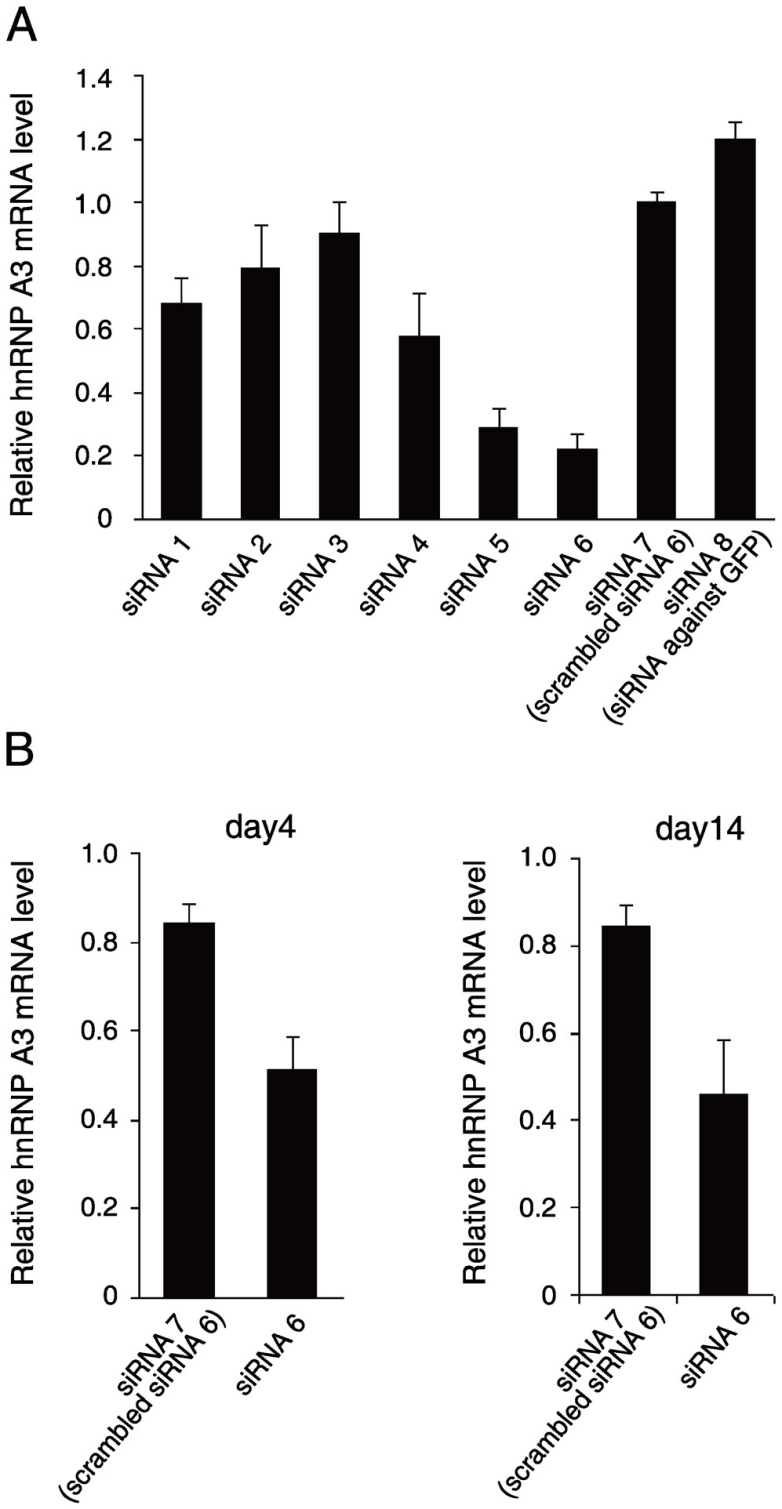
Construction of siRNAs against hnRNP A3 and their effects on hnRNP A3 expression. **A.** Suppressive effects of siRNA 1–6 (siRNA against hnRNP A3) on the hnRNP A3 mRNA level in 293T cells. Effects of siRNA were assessed in duplicates in 293T cells co-transfected with expression vector plasmids for hnRNP A3 and various siRNA expression vectors as shown in Table 1. Averages of two independent assays of each duplicated measurements per condition were normalized to siRNA 7 (scrambeled siRNA 6) for presentsation. Thin vertical lines with short horizontal bars represent ranges of the duplicated measurements. **B.** Relative hnRNP A3 mRNA levels in the mouse liver post-injection of adenoviral vectors harboring siRNA 7 and siRNA 6. Animals were injected with the adenoviral vectors harboring siRNA 7, siRNA 6 or PBS-saline (n = 4 for each condition), and on day 4 and day 14 post-injection, hnRNP mRNA levels in the liver were measured. The results are normalized to that of β-actin mRNA, and their mean relative values over that of the PBS-saline condition (no siRNA, and defined as 1) are shown. Thin vertical lines with short horizontal bars represent S.D.

Six different candidate siRNA sequence sites of the gene encoding hnRNP A3 were selected as described in the methods section (Table 1). The efficacy of each siRNA in inhibiting the expression of hnRNP A3 in 293T cells co-transfected with hnRNP A3 expressing plasmid and corresponding siRNA expression vector plasmid was analyzed in quantitative reverse transcription (RT)-polymerase chain reaction (RT-PCR) experiments (Fig. 5A). PCR primers for hnRNP A3 and GAPDH (control) used in these quantitative RT-PCR assays were 5′-GTATGGCAAGATTGAAACCATAGAG-3′ and 5′-GCCCATTAATAGTGTGGTATTTCTG -3′, and 5′-AAATGGTGAAGGTCGGTGTG-3′ and 5′-TGAAGGGGTCGTTGATGG-3′, respectively. Among the six siRNA vectors against hnRNP A3 prepared, one (siRNA 6) designed to the region, nucleotide (nt) 113 through 132, of the hnRNP A3 gene (Genebank accession number: AB201711, nt 1–1636), reproducibly reduced the hnRNP A3 mRNA level by approximately 78% in 293T cells, the largest suppression of hnRNP A3 expression (Fig. 5A), and was used for the subsequent experiments. A scrambled siRNA against hnRNP A3 (siRNA 7) (5′-acaagagcgaaccagcactt-3′, a randomized sequence of nt 113 through 132 of the hnRNP A3 gene) and a siRNA against green fluorescent protein (GFP)(siRNA 8) (Genebank accession number: AF435433.1; 5′- ggagttgtcccaattcttg-3′) were used as the controls (Table1). Upon delivering adenoviral vectors harboring siRNA 6 into the tail veins of mice (C57BL6/J), the hnRNP A3 mRNA level in the liver was lowered to 49% and 54% on day 4 and day 14, respectively, of that obtained by injection of PBS-saline (Fig. 5B).

**Table 1:** Nucleotide sequences of hnRNP A3 used as targets for constructing siRNAs and of control siRNAs.

Target sequences of siRNAs designed to hnRNP A3 and control siRNA	
**siRNA 1**	**GCAACAATCAAATTATGGA (1056–1074)**
**siRNA 2**	**GGAAGAATATAACCTGAGA (450–468)**
**siRNA 3**	**CCATAGAGGTTATGGAAGA (500–518)**
**siRNA 4**	**GGATATGGTAGCAGAAGGT (1156–1174)**
**siRNA 5**	**GCACACTTACAGACTGTGT (215–233)**
**siRNA 6**	**GCCATGATCCAAAGGAACCA(113–132)**
**siRNA 7**	**ACAAGAGCGAACCAGCACTT(scrambled siRNA6)**
**siRNA 8**	**GGAGTTGTCCCAATTCTTG (siRNA against GFP)**

Effects of transduction with adenoviral vectors harboring the most effective siRNA, siRNA 6, and its target sequence scrambled siRNA on hFIX expressions from HepG2 cells transfected in advance with -416FIX m1/1.4 and -416FIX m1, hFIX minigene expression vectors with and without AIE, respectively, are shown in Table 2. HepG2 cells transfected with -416FIXm1 and subsequently transduced with adenoviral vectors harboring siRNA 6 against hnRNP A3 or its scrambled siRNA control at multiplicities of infection (MOI) 2, 20 and 100 showed no reductions in hFIX expression over that of the cells treated with PBS-saline (control) at any MOI tested (Table 2). However, HepG2 cells transfected with -416FIX m1/1.4 and subsequently transduced with the same adenoviral vector harboring siRNA 6 showed 4, 14 and 36% reductions in hFIX expression level at MOI 2, 20 and 100, respectively, over that of PBS-saline control (*P*<0.05, Student′s *t*-test). In a similar experiment, no such reductions were observed with adenoviral vectors harboring the scrambled siRNA 6.

**Table 2:** Effects of siRNA 6 on hFIX expression from HepG2 cells transfected with -416FIXm1 (containing no AIE) and -416FIXm1/1.4 (containing AIE).

		Relative expression Activity (% ± SD)*	
siRNA	MOI	-416FIIX m1	-416FIIX m1/1.4
PBS-saline		100	100
Scrambled siRNA 6	2	105±16	100±17
siRNA 6	2	109±17	96±9
Scrambled siRNA 6	20	100±15	98±6
siRNA 6	20	109±21	86±23
Scrambled siRNA 6	100	108±23	98±16
siRNA 6	100	104±25	64±21^#^

*HepG2 cells transfected with hFIX minigene expression vector, -416FIXm1 (containing no AIE) or -416FIXm1/1.4 (containing AIE), were treated with PBS-saline, or transduced with adenoviral particles harboring scrambled siRNA 6 or siRNA 6, and hFIX protein produced was measured (n = 4). Human FIX expression activity was normalized to that of HepG2 cells treated with PBS-Saline for each expression vector condition. SD, standard deviation. #*P*<0.05 (vs. scrambled siRNA6).

### Age-related expression profiles of the hnRNP A3 gene in the mouse liver

**Figure 6:**
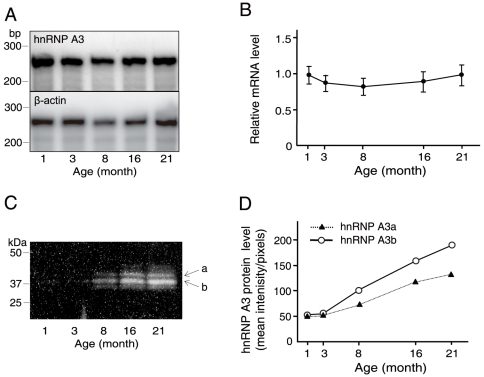
Age-dependent levels of hnRNP A3 mRNA and protein in the mouse liver. **A.** Age-related changes of the hnRNP A3 mRNA level determined by RPA. Total liver RNA preparation obtained from male C57BL6/J mice (n = 4 per age point) was subjected to quantification by RPA. The top and bottom panels are for hnRNP A3 and β-actin, respectively. Numbers at the bottom of the panels represent age of animals. Size marker positions are shown on the left. **B.** The age-related profile of the relative hnRNP A3 mRNA level normalized to that of β-actin shown in A. **C**. Western blot analysis of age-related hnRNP A3 protein levels in the mouse liver. Protein size marker positions are shown on the left, and age of animals are shown at the bottom. Two major protein bands corresponding to the known isoforms, hnRNP A3a and hnRNP A3b, are depicted with a and b with arrows on the right. **D.** The age-related increase profiles of hnRNP A3a and hnRNP A3b proteins observed in **C**.

An expression profile of the hnRNP A3 gene (mRNA level) observed for a period of 1 through 21 months of age is shown in Fig. 6A (top panel). For RNase protection assays (RPA) used in this analysis, a DNA fragment designed to protect a RNA fragment of 256 nucleoside residues in size was generated by PCR using forward and reverse primers, 5′-atggaagacaggcagagt-3′ and 5′-taatacgactcactataggagtttcctccacgaccaa-3′, respectively. A similar RPA analysis of mouse β-actin RNA (control for RPA) gave an age profile of a RNA fragment of 245 nucleoside residues in length (Fig. 6A, bottom panel). An age profile of the ratio of the protected hnRNP A3 mRNA band intensity divided by that of β-actin showed no significant age-related changes over the tested period (Fig. 6B). This was further confirmed by another RPA control, the 18S RNA fragment of 80 nucleoside residues (data not shown).

Two major hnRNP A3 protein bands of approximately 38 kDa and 41 kDa identified by Western blot analysis of the liver NEs apparently corresponded to hnRNP A3a and A3b isoforms, respectively [6], [7] (Fig. 6C). In contrast to an age-stable profile observed for the hnRNP A3 mRNA level, these protein levels gradually increased along the age-axis (Fig. 6D). Rabbit polyclonal anti-hnRNP A3 antibody used in this experiment was produced by using a mixture of two synthetic peptides of mouse hnRNP A3 sequences, FGGDGGNYGGGPGYSS (aa 270–285) and GYDGYNEGGNFG (aa 309 through 320) as antigens (see reference 6 for the aa sequence system).

## Discussion

As we previously reported [1], [2], the ASE/AIE-mediated genetic mechanism for age-related gene regulation has two essential elements, ASE and AIE. Together with other basal regulatory elements, these elements produce a unique age-related gene expression profile, and identification of their binding proteins in the nucleus remained critical for gaining insights into the age-related homeostasis mechanism. Recently, Ets1 was successfully identified as the liver protein binding to ASE [4], while identification of the nuclear proteins binding to AIE RNA remained to be achieved.

In the present study, a series of RNA EMSAs with mouse liver NEs and hFIX-AIE RNA or mFIX-AIE RNA reproducibly detected two shifted bands for both RNA probes (Fig. 1). Subsequent 2DE of a pooled sample of the UV-irradiated and RNase treated ^32^P-hFIX-AIE RNA/nuclear protein complex identified well-defined radioactive protein spots (Fig. 2). By performing repeated MALDI-TOF/MS and PMF analyses of the major radioactive protein spot, we successfully identified hnRNP A3, a hnRNP family protein [8], as a major nuclear protein binding to hFIX-AIE RNA (Fig. 3). In only one experiment out of four independent experiments (two experiments for each mFIX- and two hFIX-AIE RNA probes), hnRNP A2/B1, another hnRNP family protein, was also detected in addition to hnRNP A3. However, because of its inconsistency in binding to AIE RNA probes, a possible role of this protein in age-related regulation of gene expression is likely minor, if any. Two shifted bands reproducibly observed with AIE RNA probes (Fig. 1) presumably correspond to two known major isoforms, hnRNP A3a and A3b of 41 and 38 kDa in size, respectively (see Fig. 3) [6]–[8]. These two mouse liver isoforms of hnRNP A3 are known to have 379 and 357 aa residues, respectively. Whether or not both isoforms function equally well in age-related homeostatic regulation of genes remains to be determined. We also observed two additional bands with much weaker intensities in Western blot analyses (Fig. 6C). These may correspond to additional two minor hnRNP A3 isomers previously detected by Ma et al. [6] in Western blot analysis, but not in LC/mass-spectrometric analysis.

Both hFIX-AIE RNA and mFIX-AIE RNA, which are particularly rich in AT repeats as the common structures, specifically bind hnRNP A3 and function in a similar manner in age-related gene regulation. This is in accordance with the fact that in RNA EMSA competition assay, both homo-poly(U) and homo-poly(A) show significant competitive effects on hnRNP A3 binding to AIE RNAs, while homo-poly (C) does not, suggesting a preference of AT-rich nucleoside sequences by hnRNP A3 in its binding to AIE RNA (Fig. 1E). Although hFIX-AIE RNA, and not mFIX-AIE RNA, contains some GU and CA repeats, they appear not important in binding to hnRNP A3. In addition, we speculate that for specific binding by hnRNP A3, stem-loop structures of AIE RNAs also play an important role [5]. Further studies, however, are required for better understanding of the AIE RNA structure and hnRNP A3 binding relationship.

MALDI-TOF/MS analyses of the UV cross-linked hnRNP A3/hFIX-AIE RNA complex never detected specific peptides encompassing the region aa 100–126 (Fig. 3), while similar analyses of AIE RNA-free hnRNP A3 detected the peptides of this region (data not shown). This observation suggests that this region in the immediate neighborhood of a RNA recognition motif (RRM) known for hnRNP family proteins [12]–[14] may be a part of AIE-RNA binding site of hnRNP A3. Further studies are needed to clarify actual roles in RNA binding of this region as well as Gly-rich regions containing several Arg-Gly-Gly motifs in RNA binding, which have been previously implicated in RNA binding of various hnRNP family proteins [12]–[14].

In MALDI-TOF/MS analyses of the nuclear hnRNP A3 in complex with the hFIX-AIE RNA, a mass-spectrum *m/z* 1910.9, which corresponds to the peptide aa 356–377 with its Ser^359^ unphosphorylated, was detected, while *m/z* 1990.8 corresponding to the same peptide with its Ser^359^ phosphorylated was always absent (Fig. 4, A and B). In contrast, RNA fragment-free liver nuclear hnRNP A3 reproducibly gave both *m/z* 1910.9 and *m/z* 1990.8, indicating that the Ser^359^ residue is in both states of partially unphosphorylated and phosphorylated (Fig. 4C). Here, *m/z* 1910.9 detected could be in part due to a partial release of the phosphate group from the once phosphorylated Ser^359^ residue in the fragmentation process of the protein in MALD-TOF/MS analysis. The phosphorylated status of Ser^359^ is of particular interest, because in analogy to other hnRNP proteins [11], [15]–[20], phosphorylation and dephosphorylation of this region of hnRNP A3 likely play critical roles in regulation of FIX mRNA translation and also intracellular recycling of hnRNP A3 itself. In a global analysis, Villen et al. [20] found that Ser and Tyr residues in the region (aa 356–377) of hnRNP A3 including Ser^359^ are phosphorylated. In the present study, however, we did not obtain any evidence for phosphorylation of Ser and Tyr residues other than Ser^359^ (Fig. S1). Yamaoka et al. [11] reported that in the cells transformed with temperature-sensitive Rous sarcoma virus, the level of phosphorylated human hnRNP A3 at one or more of the three serine residues present in the region aa 355 through 376 (equivalent to aa 356–359 in mouse hnRNP A3 due to the presence of an extra aa residue G^309^) increased with temperature. HnRNP K, one of the hnRNP family proteins, is known to bind to the differentiation control element (DICE), a CU-rich stretch of sequence in the 3′-UTR of 15-lipoxygenase (LOX) mRNA, inducing translational repression of the LOX gene [16]. This repressive effect is removed upon tyrosine phosphorylation of the protein [17]. Zipcode binding protein 1 (ZBP1), which binds to a conserved 54-nucleotide region in the 3′-UTR of β-actin mRNA, prevents the mRNA from being used for translation, but again like hnRNP K, upon phosphorylation of Tyr^396^, it frees the bound mRNA, allowing it to be used for translation [19]. Similar to other hnRNP family proteins, hnRNP A3 is presumably regulated not only by phosphorylation but also by various other types of modifications including methylation [21], [22] and sumoylation [23]. These various protein modifications in addition to alternative splicings apparently give rise to many hnRNP A3 protein spots detected in 2DE.

Our efforts for constructing effective adenoviral vectors harboring siRNAs against hnRNP A3 resulted in identification of a highly effective siRNA, siRNA 6, which was capable to reduce the hnRNP A3 mRNA level by 78% in 293T cells (Fig. 5A). This siRNA also lowered the hnRNP A3 mRNA level in mice to 49% and 54% of that obtained with PBS-saline control on day 4 and day 14, respectively (Fig. 5B). We then tested this construct for its effects on hFIX expression from HepG2 cells transfected with -416FIX m1, a hFIX expression vector without AIE, and -416FIXm1/1.4, a hFIX expression vector containing AIE. These hFIX expression vectors were previously characterized extensively for their stable hFIX expression at substantial levels in HepG2 cells [1]. Transduction of HepG2 cells carrying -416FIX m1 with and without siRNA 6 containing adenovirual particles at various MOIs showed no reduction in hFIX expression at all conditions tested (Table 2). In a similar experiment, however, hFIX expression from the HepG2 cells carrying -416FIXm1/1.4 showed siRNA-specific and adenoviral MOI-dependent reductions in hFIX expression, while no such reduction in expression was observed with the cells transduced with scrambled siRNA 6 containing adenoviral particles (Table 2). Together, these observations further support the critical roles of AIE and its binding protein, hnRNP A3, in hFIX expression.

The hnRNP A3 mRNA level in the mouse liver stays stable throughout the tested period of age (1 through 21 months) (Fig. 6A and B). In contrast, the concentration level of hnRNP A3 protein in the liver gradually increases in an age-dependent manner (Fig. 6C and D). This discordance in the age-related expression patterns of mRNA and protein of hnRNP A3 indicates that hnRNP A3 is under regulation of age-dependent epigenetic events. This is different from what we observed for HuR, an AU-rich RNA domain binding protein [24], showing age-stable expression patterns for both mRNA and protein (data not shown). Further detailed studies on the age-related regulation of gene by hnRNP A3 with the siRNA using an animal model and underlying mechanism of action of hnRNP A3 still remain to be done.

HnRNP A3 is a recently found member of the hnRNP family protein, currently composed of more than 20 different proteins including hnRNP A1, A2, B1 and B0a/b, which are known to have highly diverse functions such as precursor mRNA processing, transcriptional regulation and DNA recombination [6]–[8], [25]. HnRNP A3 has been reported to bind to the telomere sequence, likely playing an important role in its maintenance [26], and to the 3′-UTR region of the cyclooxygenase-2 gene, playing a role in lipopolysaccharide signal transduction [10]. This protein is also known to bind to the AU-rich element in the 3′-UTR region of TNFα gene, likely working as an inflammatory mediator [9].

This study has successfully assigned a novel functional role to hnRNP A3 in age-related regulation of gene expression, opening up a new avenue for studying age-related homeostasis and underlying molecular mechanisms, and for developing new preventive methods and treatments for relevant age-associated diseases.

## Materials and Methods

### Materials

Radiolabeled nucleoside, α [^32^P]-UTP, and poly(U), poly(A) and poly(C) homoribonucleoside oligomers were obtained from GE Healthcare (Little Chalfont, UK). Antibodies used for hFIX-specific enzyme-linked immunosorbent assay (ELISA) and HepG2 cell line were obtained as previously described [1], [27].

### Animals

Mice (C57BL/6J) at various ages were obtained from Charles River Laboratories (Wilmington, MA). Animal care and use were reviewed and approved by the Committee for Animal Experimentation of the National Institute of Advanced Industrial Science and Technology (AIST)(permission # 36-07-009) and were performed in accordance with the institutional guidelines of the Committee for Animal Experimentation in the AIST.

### Construction of AIE RNA probes

^32^P-labeled AIE RNA probes were prepared by using T7-MEGAshortscript high yield kit according to the manufacture's instruction (Ambion, Austen, TX). *In vitro* transcription was done for 2 h at 37°C with a template DNA fragment of 149 bps, harboring hFIX-AIE region (102 bps). This template DNA fragment was prepared by PCR using the human genomic DNA as a template. Forward and reverse primers of 5′-taatacgactcactatagggaagtttctttcagagagttaagtta-3′ comprised of a T7 promoter sequence (20 bps) and a 5′ 25 nucleotide portion of the hFIX-AIE region spanning from nt 32119 through 32143 [5], and 5′-tagaatggcttattgcttccattatatgt-3′(nt 32239–32267), respectively, were used. The template DNA fragment (149 nucleoside residues in length: 5′-aagtttctttcagagagttaagttattttatatatataatatatatataaaatatataatatacaatataaatatatagtgtgtgtgtgtatgcgtgtgtgtagacacacacgcatacacacatataatggaagcaataagccattcta-3′) was then transcribed, generating the corresponding hFIX-AIE RNA. Mouse FIX-AIE RNA (50 nucleosides in length: 5′-gaaatcattaatttaatcatattggtaatatatatatattatatctctaa-3′) was produced by using a chemically synthesized DNA fragment, 5′- taatacgactcactatagggaaatcattaatttaatcatattggtaatatatatatattatatctctaa-3′, comprised of a T7 promoter sequence (19 bps) and the mFIX-AIE sequence (50 bps) spanning a region nt 2056 through nt 2105 of the mFIX gene (Genebank accession number: M23109). Transcriptional reaction with each of these AIE DNA templates was carried out in a reaction mixture (final volume of 20 µl) composed of 2 µl of 10x transcription buffer provided in the Ambion kit, 2 µl of each 75 mM ATP, GTP, CTP, 4 µl α [^32^P]UTP (GE Healthcare, 800 Ci/mmol), 1 µg hFIX-AIE or mFIX-AIE template DNA and 2 µl of T7 MEGA shortscript enzyme mix. The reaction mixture was then added with 1 µl of RNase-free DNase solution, incubated for 15 min at 37°C and subjected to electrophoresis using a 6% polyacrylamide urea gel. The gel was then exposed to an X-ray film for 10 sec, and gel areas containing radioactivity were precisely located by matching with the autoradiogram and excised. The gel pieces recovered were then incubated in a probe elution buffer (Ambion) at 37°C overnight. Non-radioactive AIE RNA probes were prepared in a similar manner by using non-radioactive nucleosides in transcription reaction.

### Electrophoretic mobility shift assay (EMSA) with ^32^P-AIE RNA

EMSA with an AIE RNA probe was carried out as described by Cok et al. [10] with some modifications. NEs were prepared from the mouse liver tissues (C57BL/6J mice) at 6 months of age as previously described [1]. Aliquots (20,000 cpm) of ^32^P-AIE RNA probe with a specific activity of approximately 1.9×10^9^ cpm were incubated for 50 min on ice with 2.5, 5 or 10 µg of NEs in the presence of 0.2 µg of yeast RNA and 15 U RNase inhibitor in EMSA binding buffer (10 mM Tris-HCl, 50 mM NaCl, 5% glycerol, 1 mM DTT, 1 mM EDTA). The reaction mixture was then added with 8 µl loading buffer, and subjected to EMSA on a 10% non-denaturing polyacrylamide gel. The gel was scanned with a Storm 860 PhosphorImager (GE Healthcare). For competitive EMSAs, non-radiolabeled RNA probe in various molar excesses over that of ^32^P-labeled RNA probe was added to the reaction mixture containing NEs 5 min prior to addition of the radiolabeled probe. The rest of EMSA was performed as described [10].

### UV cross-linking, RNase-treatment and analysis by sodium dodecylsulfate polyacrylamide gel electrophoresis (SDS-PAGE) of ^32^P-hFIX-AIE RNA/nuclear proteins complex

UV-crosslinking, RNase treatment and analysis by SDS-PAGE of ^32^P-hFIX-AIE RNA/nuclear proteins complex were performed as follows. Liver NEs (10 µg) was incubated with ^32^P-hFIX-AIE RNA probe **(**approximately 1×10^6^ cpm/10 µl**)** in a similar condition as described above. The reaction mixture was then exposed to UV light for 30 min on ice (UV Stratalinker 1800, Stratagene), followed by incubation with 2 µl of RNase T1 (5 U/µl) (Ambion) for 20 min at 25° C. The reaction mixture was then added with 8 µl SDS loading buffer (0.125 M Tris-HCl, 4% SDS, 4% 2-mercaptoethanol, 0.02% BPB, 20% glycerol), and subjected to SDS-PAGE using a 10% polyacrylamide gel in the presence of 0.1% SDS. SDS-PAGE gels were scanned with a Storm 860 (GE Healthcare). For competitive RNA EMSAs, non-radiolabeled hFIX-AIE or mFIX-AIE RNA probe in various molar excesses over that of ^32^P-labeled hFIX-AIE or mFIX-AIE RNA was added to the reaction mixture containing NEs 5 min prior to addition of the radiolabeled AIE RNA probe. The rest of conditions including UV cross-linking, RNase T1 treatment and SDS-PAGE was same as above. Competition assays with 10 µg each of homo-poly nucleoside fragments, homo-poly(U), homo-poly(A) and homo-poly (C), were done in a similar manner as for non-radiolabeled AIE RNA competitors.

### Preparative isolation of nuclear protein/^32^P-hFIX-AIE RNA complex by solution-phase isoelectric focusing (IEF) and two dimensional electrophoresis (2DE)

For identification of the hFIX-AIE RNA binding protein(s), preparative RNA EMSA using 20 µg NEs was repeated in a similar condition as for analytical scale RNA EMSA. Following the UV cross-linking step, the reaction mixture was added with 2 µl RNase T1 (5 U/µl) and incubated for 20 min at 25° C. The reaction mixture was subjected to a preparative SDS-PAGE using NuPAGE® Novex Bis-Tris 4–12% ZOOM® Gel (Invitrogen, Carlsbad, CA). The gel was exposed to an X-ray film at 4°C for 1 day. Radioactive areas of the gel containing the nuclear protein/^32^P-AIE RNA complex were excised and subjected to electro-elution using an Amicon Centriruter microelectroeluter (Millipore, Billerica, MA). The solution recovered was concentrated with a Centricon apparatus (Millipore). This procedure was repeated to obtain a sufficient amount of nuclear protein/^32^P-labeled hFIX-AIE RNA complex for further analyses. The pooled sample solution was then subjected to IEF using a ZOOM® IEF Fractionator (Invitrogen), and solution fractions with radioactivity were recovered, concentrated with a Centricon apparatus and subjected to analytical 2DE. Its IEF (the first dimension) was performed by using an IPG Runner system with a pre-casted ZOOM strip of an appropriate pH range gradient, followed by SDS-PAGE using ZOOM gels. The gel was then silver stained and exposed to an X-ray film at 4°C for 1 to 6 days depending on the radioactivity strength. For identification of a protein(s) bound to the AIE RNA probe used, the central gel area of radioactive spots visualized in autoradiogram was excised and in-gel digested with trypsin (Promega, Madison, WI). Peptides generated were then recovered by extracting with a solution of acetonitrile:water:trifluoroacetic acid (66:33:0.1 by volume), vacuum-concentrated and cleaned with a Zip Tip C18 (Millipore). The peptide containing fractions collected and dried were mixed with CHCA matrix before applying to an Axima® CFR MALDI-TOF/MS (Shimazu Biotech, Kyoto, Japan). PMF analysis was done with a MASCOT server version 2.1 (Matrix Science, London, U.K.) taking the UniProtKB mouse protein database (taxonomy: mus musculus).

### 2DE analysis of mouse liver nuclear proteins

Mouse liver NEs (300 µg) were subjected to IEF using a 24 cm immobiline DryStrip (pH 6–11) (BIO-RAD, Hercules, CA), followed by SDS-PAGE using a pre-casted 10–16% gradient polyacrylamide gels (BIO-RAD). Gels were then stained with Colloidal Coomassie Brilliant Blue (CBB) and scanned by GS-800 scanner (BIO-RAD). Protein spots were analyzed with a PDQuest software (BIO-RAD, version 7.1), and excised, destained in 50 mM ammonium bicarbonate/50% acetonitrile and dried in a vacuum concentrator (Savant, Holbook, NY). Dried gel pieces were then rehydrated with 5 µl of 20 ng/L trypsin in 10 mM ammonium bicarbonate, and incubated at 30°C overnight. Mass-spectrometric analyses of the tryptic peptide samples were carried out as described above except by using an AXIMA® MALDI-TOF2 (Shimadzu Biotech).

### Construction of recombinant adenovirus harboring siRNA

Six separate candidate sequence sites of the AIE RNA binding protein gene were selected by utilizing the siRNA design support system R3 program (Takara Bio, Inc., Shiga, Japan) for constructing an optimal siRNA expression unit against the gene. Double stranded (ds) 19 or 20 nt-long sequences corresponding to these sites were synthesized by mixing complementary oligonucleotides containing a loop sequence (ctgtgaagccacagatggg) with BamH1 and Hindzш sticky ends at 5′ and 3′ ends, respectively, thus forming a short hairpin RNA against the protein RNA. The expression units encoding siRNAs against to the target protein were cloned into pBAsi-mU6 Neo DNA vector (Takara). The efficacy of each siRNA was assessed in 293 cells co-transfected with the target binding protein expressing plasmid (0.1 µg) and its siRNA expressing plasmid (0.3 µg). An expression vector unit for the target protein, was prepared by RT-PCR with the total mouse liver RNA as a template, followed by insertion into the Gateway® pDESTTM 26 Vector using the Mammalian Expression System with Gateway® Technology kit (Invitrogen). All measurements of each siRNA test sample were done in duplicated RT-PCR. After subtracting the PBS-saline control background, the results were normalized to that of GAPDH (control), and averaged**.** The most effective siRNA identified in suppressing the target protein mRNA level was then cloned into pBAsi-mU6 Neo vector, and digested with EcoRV, thus releasing a siRNA-encoding fragment. The fragment was then inserted into a cosmid pAxcwit vector (Takara Bio) at a SwaI site, and the cosmid vector generated was used for producing recombinant adenoviral particles harboring the highly effective siRNA expression unit with a Takara adenovirus expression vector kit. Adenoviral particles produced were purified by CsCl gradient centrifugation and stored at −80°C until use. Control recombinant adenoviral particles harboring a scrambled siRNA of the above selected siRNA or a siRNA against GFP gene were similarly constructed.

Recombinant adenoviral particles harboring siRNA expression unit against the target protein ranging from 8.4×10^9^ pfu in 100 µl PBS were administered into tail veins of mice (C57BL6/J) at 12–17 month of age (n = 4 for each experimental condition). Animals were then sacrificed on day 4 and 14 post-viral injection, their liver tissues removed and quick frozen on dry ice, and stored at –80°C until use. Total RNAs were prepared from these tissues by a procedure of acid guanidium phenol chloroform. PCR primers and TaqMan probe used for the target protein in these quantitative real-time RT-PCR assays were 5′-TGTCAGGAAAGCTGCAGGTTACT-3′ and 5′- CATCGTTCCTTCTGTGGCAGATT-3′, and 5′-FAM-CATCGTGCACCGCAA-MGB-3′, respectively. Similarly, for β–actin (control), 5′-TCCACCTTCCAGCAGATGTG-3′ and 5′-CAGTAACAGTCCGCCTAGAAGCA, and 5′-FAM-CATCGTGCACCGCAA-MGB-3′ were used, respectively. RT-PCR Results were normalized to that of β–actin mRNA.

### Analysis of siRNA effects on hFIX expression in HepG2 cells

HepG2 cells were cultured in Dulbecco's modified eagle medium (DMEM) as previously described. The cells were transfected with a mixture of hFIX minigene expression vector, -416FIXm1/1.4 (hFIX expression vector containing AIE) or with -416FIXm1 (hFIX expression vector without AIE) [1], and pCH110 (β-galactosidase expression vector) at a 10∶1 (w/w) ratio (total 3 µg/well of 6-well plates) with Fugene 6 (Roche Diagnostics) as previously described [1]. After 1 hr of transfection reaction, the cells were added with recombinant adenoviral particles (1.7×10^7^ pfu/µl in PBS-saline) at 2, 20 and 100 MOI, and incubated at 37°C for 1 hr under gentle rocking every 10 min. The medium was then switched to fresh DMEM and incubation continued for 48 hr. Human FIX produced to the medium was then quantified by hFIX-specific ELISA as previously described [1].

### Ribonuclease protection assay (RPA)

RPA for quantifying the mRNA level of hFIX-AIE RNA binding nuclear protein was performed using a RPAш kit (Ambion) according to the manufacturer′s instruction. Total RNAs of liver tissues of mice (C57BL6/J) at various ages (n = 4 per age point) were prepared by an acid guanidium phenol chloroform procedure [28]. The antisense DNA probe of the protein mRNA was prepared by PCR amplification. Mouse β-actin DNA (pTRI-Actin) provided in a RPAш kit and 18S DNA (pTRI-RNA, Ambion) were used as the controls in RPA. Antisense ^32^P-labeled cRNA probes were generated using a MEGAscript *in vitro* transcription kit (Ambion) and α [^32^P]-UTP. Hybridization was performed by incubating an aliquot of total liver RNAs (10 µg) with ^32^P-labeled probe (approximately 1×10^6^ cpm/10 µl) for overnight at 42°C. The resulting hybridized mixture was digested with a mixture of RNase A (2.5 units/ml) and RNase T1 (100 units/ml) in RPAш kit. The final precipitates obtained were then dissolved in gel loading buffer, and subjected to SDS-PAGE using a 6% polyacrylamide gel with 8M urea. The gel was transferred onto a Whatmann 3 MM paper, dried and exposed to an imaging plate (Fuji Film, Tokyo, Japan). The plate was then scanned and quantified for radioactivity with a Storm 860 (GE Healthcare) using a NIH imaging program.

### Western blot analysis

Liver NEs (10 µg/assay) prepared as described above from C57BL/6J mice at various ages (n = 10 per age point) were added with 20 µl SDS loading buffer, and its protein concentration was measured by the BCA method [29] and appropriately adjusted with the loading buffer for SDS-PAGE using a 10% gel. The rest of Western blotting procedure was performed according to the standard method using horseradish peroxidase-conjugated anti-rabbit antibody for protein detection with the ECL assay system (GE Healthcare) [30]. Quantification of bands was done by using a Bio-imaging analyzer LAS 1000 with Image Gauge Ver. 3.4X software (Fuji film).

## Supporting Information

Figure S1 MS/MS analysis of the peptide fragment from a mass peak of m/z 1990.8. The fragmentation spectra of the peptide (aa 356 through 377, SSGSPYGGGYGSGGGGSGGYGSR) of mouse hnRNP A3 are shown at the lower panel. Annotated ions as nominal m/z values and results from fragmentation of an amide bond with charge retention on the carboxyl end (y series ion) or amino terminus (b series ion) are shown. Mascot scores for possible phosphorylation site at Ser 359, Ser 357, Ser 356, Ser 367 are 138.6 (Expect value, 1.1 e-12), 125.8 (2.1 e-11), 123.4 (3.7 e-11), 38.2 (0.012), respectively. These data indicated that Ser 359 is the most likely phosphorylation site. Fragment ions, y19-98, y20-98, b4-98, b5-98, b9-98, b10-98, b13-98, b19-98 and b21-98 showed a loss of 98-Da in mass corresponding to a phosphate group. Fragment ions b2 and y18 indicated that a phosphate group is not present in the SS dipeptide sequence (Ser 356 through Ser 357) and PYGGGYGSGGGGSGGYGSR (Pro 360 through Arg 377).
(1.30 MB EPS)
